# Tuning the Mechanical and Antimicrobial Performance of a Cu-Based Metallic Glass Composite through Cooling Rate Control and Annealing

**DOI:** 10.3390/ma10050506

**Published:** 2017-05-06

**Authors:** Victor Manuel Villapun, Faye Esat, Steve Bull, Lynn George Dover, Sergio Gonzalez

**Affiliations:** 1 Faculty of Engineering and Environment, Northumbria University, Newcastle upon Tyne NE1 8ST, UK; victor.puzas@northumbria.ac.uk; 2 School of Chemical and Process Engineering, University of Leeds, Leeds LS2 9JT, UK; F.Esat@leeds.ac.uk; 3 Newcastle University, School of Chemical Engineering and Advanced Materials, Newcastle upon Tyne NE1 7RU, UK; steve.bull@ncl.ac.uk; 4 Faculty of Health and Life Sciences, Northumbria University, Newcastle upon Tyne NE1 8ST, UK; lynn.dover@northumbria.ac.uk

**Keywords:** tribological properties, antimicrobial behaviour, metallic glass composite, scratch test, scratch hardness

## Abstract

The influence of cooling rate on the wear and antimicrobial performance of a Cu_52_Z_41_Al_7_ (at. %) bulk metallic glass (BMG) composite was studied and the results compared to those of the annealed sample (850 °C for 48 h) and to pure copper. The aim of this basic research is to explore the potential use of the material in preventing the spread of infections. The cooling rate is controlled by changing the mould diameter (2 mm and 3 mm) upon suction casting and controlling the mould temperature (chiller on and off). For the highest cooling rate conditions CuZr is formed but CuZr_2_ starts to crystallise as the cooling rate decreases, resulting in an increase in the wear resistance and brittleness, as measured by scratch tests. A decrease in the cooling rate also increases the antimicrobial performance, as shown by different methodologies (European, American and Japanese standards). Annealing leads to the formation of new intermetallic phases (Cu_10_Zr_7_ and Cu_2_ZrAl) resulting in maximum scratch hardness and antimicrobial performance. However, the annealed sample corrodes during the antimicrobial tests (within 1 h of contact with broth). The antibacterial activity of copper was proved to be higher than that of any of the other materials tested but it exhibits very poor wear properties. Cu-rich BMG composites with optimised microstructure would be preferable for some applications where the durability requirements are higher than the antimicrobial needs.

## 1. Introduction

The emergence and transmission of antimicrobial resistance in bacterial populations in recent years is of major concern and represents a serious threat to global public health. The World Health Organization is coordinating a global action plan to address the issues that have contributed to the dawn of a post-antibiotic era in which once easily managed infections might be no longer adequately controlled, with significant human and socioeconomic consequences [1]. This is especially challenging in hospitals, where patients are often immunocompromised by a number of diseases and their treatments, thus making them more vulnerable to infections.

Despite concerted efforts in preventing nosocomial infections (i.e., hospital-acquired infections), the total annual number of patients with healthcare-associated infections (HAI) in European hospitals was around 3.2 million in 2011–2012 [2,3,4,5], while in the USA approximately 2 million patients suffer from a HAI, of whom about 90,000 die per year [6].

An important source of HAI is hospital touch surfaces, which are contaminated via contact with human skin. Hands are commonly colonised by a complex mixture of bacteria, i.e., more than 150 species have been identified [7]. Some of these bacteria can be potentially hazardous [8] and can be transmitted through surfaces such as bedrails, resulting in infections spreading.

A useful preventative measure to limit transmission of infections from touch surfaces is the use of disinfectants [9]. In order to efficiently disinfect surfaces using these chemical products, new recommendations on disinfection have been developed [10,11]. The major drawbacks of using a disinfectant cleaning regime are the temporary effectiveness and the dependency on the thoroughness of the operator. A safer option might be the use of materials with antibacterial properties; their efficacy in reducing HAI rates has been recognised in the last decade [12,13].

Numerous antimicrobial studies have been carried out over the years on elements that are traditionally regarded as antibacterial (e.g., copper, zinc and silver) [14,15,16]. Among these metals, copper has been widely used for healthcare applications due to its high performance in eliminating bacteria and relatively low cost [17,18,19,20]. As a result, the interest in developing copper-based alloys for touch surfaces, such as door plates, has been increasing over the last few years as well as their implementation in hospitals. These touch surfaces may also be subjected to wear due to contact with bed rails, etc., and therefore durability is also an important property to take into consideration. In this regard, bulk metallic glasses (BMGs) are promising candidate materials due to their hardness, corrosion and wear resistance [21]. Additionally, BMGs can display bactericidal activity when containing a high concentration of antimicrobial elements such as Cu (i.e., Cu-rich alloys) [22,23]. However, the lack of grain boundaries in BMGs may limit the diffusion of copper ions and therefore decrease the killing performance when compared to crystalline materials [23,24]. The development of Cu-based BMG composites with optimum control of the composition combines high bactericidal performance and wear resistance [25]. However, an important question that remains open is whether changing the cooling rate instead of the composition can be an effective route for tuning the antimicrobial and mechanical performance of Cu-based BMG composites. To explore this issue, a Cu-rich BMG composite with very low Cu content (i.e., Cu_52_Z_41_Al_7_ (at. %)) has been explored since for low Cu contents a minimum antimicrobial behaviour is expected and therefore this would correspond to the lower bound. The performance of the BMG composite will be compared with that of a material of the same composition but fully crystalline (after annealing at 850 °C for 48 h) [26,27,28,29,30].

Another novelty of this manuscript is the analysis of the standardisation of antimicrobial activity by comparing the different methodologies (European, American and Japanese). To do so, the antimicrobial activity of the Cu-based BMG samples obtained at the highest (i.e., CH 2 mm) and lowest (i.e., NCH 3 mm) cooling rate will be compared. The lack of standardisation of antimicrobial susceptibility testing results in different methodologies makes it difficult to compare data. As far as the authors are concerned, only three bactericidal activity standards of hard surfaces have been reported: the Japanese “Antibacterial products-Test for antibacterial activity and efficacy” [31], the European “Plastics-Measurement of antibacterial activity on plastic surfaces” [32] and the American “Protocol for the evaluation of bactericidal activity of hard, non-porous Copper containing surface products” [33]. However, the European standard is based on the Japanese standard and therefore only two different methods for calculating the bactericidal effect will be explored in this study (i.e., the Japanese and the American). Both standards are mostly focused on the previously mentioned bacteria (*S. aureus* and *E. coli*) for JIS Z 2801:2010 and (*S. aureus* and *P. aeruginosa*) for the American protocol. At the same time, it has to be mentioned that both standards provide protocols and equations to accurately estimate the bactericidal activity on the analysed substrate. Nevertheless, only the EPA protocol gives a threshold to consider a material sanitary. This protocol dictates that a material that is able to eliminate 99.9% of bacteria after 1 h of contact time can be labelled as such. In this paper, a variation of the EPA protocol will be used and the bactericidal effect of the metallic glass composites studied will be calculated using the guidelines of both standards.

The main aim of this work is to assess the feasibility and effectiveness of tuning the mechanical and antimicrobial performance of Cu_52_Z_41_Al_7_ (at. %), a Cu-based BMG composite of low Cu-content, through cooling rate control. In order to analyse the effect of the microstructure and grain boundaries on the performance the BMG composite will be annealed to 850 °C for 48 h. This work will also provide an analysis of the standardisation of antimicrobial activity using different methodologies.

## 2. Materials and Methods

Alloy ingots of nominal composition Cu_52_Zr_41_Al_7_ were prepared from elements with purity higher than 99.9 at. %. The master alloys were re-melted three times in a Zr-gettered high purity argon atmosphere to attain good chemical homogeneity. Rod samples of 2 mm and 3 mm in diameter were obtained from the master alloy by copper mould casting in an inert gas atmosphere at two different conditions, with the cooling system on (10 °C) and off (20 °C). From now on the chilled sample will be named CH and the non-chilled sample NCH. A NCH 3 mm Cu_52_Zr_41_Al_7_ rod was annealed at 850 °C for 48 h in a furnace in air to study the effect of a fully crystalline microstructure on the antimicrobial and mechanical performance. An increase in diameter as well as changes in the cooling system temperature will decrease the cooling rate of the casted metal. As the cooling rate decreases, the ratio of crystalline to amorphous phase increases, making possible the analysis of different metallic glass composites. Additionally, a pure copper sample was used as a reference for assessing the performance of the Cu_52_Zr_41_Al_7_ alloy. All samples were stored in a sealed desiccator with self-indicating silica gel. Tests were performed during the next three months after casting. No changes in the tonality of the indicator were observed during the storage and testing of the samples.

The structure of the samples was studied by X-ray diffraction (XRD) using a Bruker D8 diffractometer (Bruker, Banner La, United Kingdom) with monochromated Cu Kα_radiation (2θ range 20°–90°, step size = 0.03°). The microstructure was investigated using a scanning electron microscope (SEM) (Mira 3 FEM-SEM, TESCAN, Cambridge, United Kingdom) equipped with energy-dispersive X-ray (EDX) analysis (results given from now on in at. %). A Teer Coating Limited scratch tester model ST220 (Teer coatings Ltd, Worcester, United Kingdom) fitted with a Rockwell C diamond stylus (cone angle 120°, radius of spherical tip 200 µm) was used for the scratch tests. The tests were performed at a load of 30 N at a ramp rate of 10 mm/min. Profiles prior to testing and the scratch track after testing were measured using an Alicona profilometer (InfiniteFocus, Alicona UK, Sevenoaks, United Kingdom). Reported data is the average of 10 measurements. Prior to the scratch tests, the surfaces were polished until the surface exhibited a mirror-like appearance by using 1 µm diamond paste.

To assess the wear resistance of this alloy, scratch hardness numbers were calculated in accordance to ASTM “Standard test method for scratch hardness of materials using a diamond Stylus” [34]. This parameter has been used successfully to analyse the wear performance of metals [35], polymers [36], ceramics [37], and coatings [38]. This parameter was calculated from the scratch width using the following equation:

(1) $$H_{s}=q\frac{4*P}{\pi *w^{2}},$$

where *H_S_* [Pa], *P* [N] and *w* [m] refer to scratch hardness, normal load and scratch width respectively and *q* is a constant dependent on the material mechanical response and how it supports the indenter (*q* ≈ 2 for rigid plastic behaviour and 1 < *q* < 2 for viscoelastic plastic materials) [39]. In this paper, the material response has been approximated as a rigid plastic and therefore we have taken *q* = 2, which is consistent with that previously reported in metallic composites [40]. Contact angle measurements were carried out using the sessile drop technique, with a Krüss drop size DSA30 analyser and depositing 1 μL of deionised water at a rate of 30 μL/min. Previously to the sessile tests, samples were gently dry polished using several abrasive silicon carbide grinding paper of lower grit size to a minimum of P4000, and cleaned in 100% ethanol in an ultrasound bath. Contact angle results are the average of five sessile drop tests (ten contact angle measurements).

For initial antimicrobial tests, *Escherichia* coli strain K12 (Gram-negative) was incubated in an orbital incubator (37 °C, 200 rpm), in 25 mL of LB (*Luria Bertani*) broth for 16 h. Culture yield was quantified by measuring optical density and the bacteria were then diluted in sterile LB Broth to an optical density (OD_600_) of 0.01. The diluted cultures were incubated at 37 °C until they reached an OD_600_ ~ 0.3. Samples were gently dry polished using several abrasive silicon carbide grinding paper discs of lower grit size to a minimum of P4000. Polished samples and control samples (stainless steel) were immersed in 100% ethanol and sonicated for 5 min in an ultrasound bath to ensure a clean and disinfected surface. The disinfected samples were left to dry in a sterile petri dish. These samples were placed inside a sterile petri dish containing tissue paper wetted with 1 mL of sterile LB Broth to prevent sample drying. A quantity of 1 µL of these cultures was dispensed directly onto the grounded surfaces (4000 grit) and control (stainless steel), and incubated at room temperature in the sealed containers. After the designated exposure time, the samples were diluted in 99 µL of Tween 20 0.148 g/L (2 × CMC) and sonicated for 5 min. Finally, the recovered bacterial suspension was subject to serial decimal dilution, and samples were spread onto LB agar plates and resulting colonies were counted after 16 h of incubation at 37 °C. All tests were performed five times, with mean counts and standard deviation reported.

## 3. Results and Discussion

### 3.1. Microstructure

**Figure 1:**
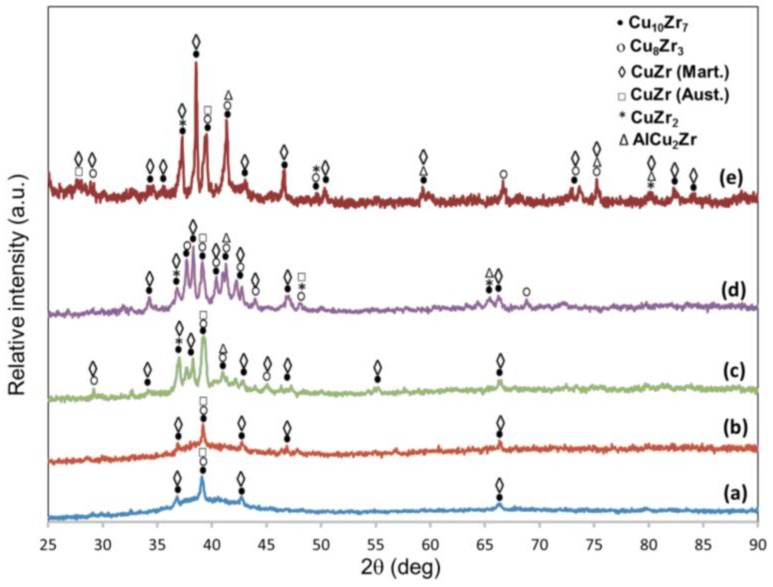
XRD scans for Cu_52_Zr_41_Al_7_ at. % alloy (**a**) CH 2 mm diameter; (**b**) NCH 2 mm diameter; (**c**) CH 3 mm diameter; (**d**) NCH 3 mm diameter; (**e**) 3 mm annealed at 850 °C for 48 h.

Figure 1 shows the XRD scans for Cu_52_Zr_41_Al_7_ at. % rods of 2 and 3 mm diameter cast chilled (CH), non-chilled (NCH) and annealed. The 2 mm diameter samples (Figure 1a,b) exhibit high intensity peaks arising from orthorhombic Cu_10_Zr_7_ (a = 0.9347 nm, b = 0.9313 nm, c = 1.2675 nm), orthorhombic Cu_8_Zr_3_ (a = 0.7868 nm, b = 0.8146 nm, c = 0.9977 nm), cubic austenite B2 CuZr (a = 0.3256 nm, b = 0.3256 nm, c = 0.3256 nm) and monoclinic martensite B19´ CuZr (a = 0.3237 nm, b = 0.4138 nm, c = 0.5449 nm). These sharp peaks are superimposed on a broad halo, suggesting the presence of an amorphous matrix. The intensity of this halo decreases slightly for the NCH sample for which an additional XRD peak is detected, thus suggesting a decrease in volume fraction of amorphous matrix for the NCH sample.

The XRD peaks of the 3 mm diameter samples (Figure 1c,d) can also be attributed to the same crystalline phases but additionally, very small peaks associated with tetragonal CuZr_2_ (a = 0.3220 nm, b = 0.3220 nm, c = 1.1183 nm) are also present. These peaks are more clearly detected for NCH 3 mm than for CH 3 mm, which is consistent with the difference in cooling rates. The number of peaks and their intensity are much higher than those detected in the 2 mm diameter samples, while the intensity of the broad halo is smaller than in the 2 mm diameter sample, thus suggesting that the alloys are more crystalline. For the CH 3 mm diameter sample, the intensity of the large XRD peak at about 39.3° and associated to Cu_10_Zr_7,_ Cu_8_Zr_3_ and austenite B2 CuZr and it is of higher magnitude than the peaks for the 2 mm diameter samples. Additionally, there is an intensity increase of the peaks detected at about 38.3° and 41.2°. Figure 1e shows the XRD scan of a 3 mm diameter sample annealed at 850 °C for 48 h. This scan does not reveal any halo associated with an amorphous matrix, thus revealing a complete crystalline microstructure. The intensity and variety of the XRD peaks are higher and the microstructure is more complex than in the previous samples. Most of the peaks correspond to the previously mentioned structures with higher intensities (especially for the peaks corresponding to Cu_10_Zr_7,_ B19´ CuZr and CuZr_2_). At the same time, it is interesting to notice the intensity of the peak at 41.3°. This peak and others found in Figure 1e are associated with cubic AlCu_2_Zr (a = 0.6190 nm, b = 0.6190 nm, c = 0.6190 nm).

**Figure 2:**
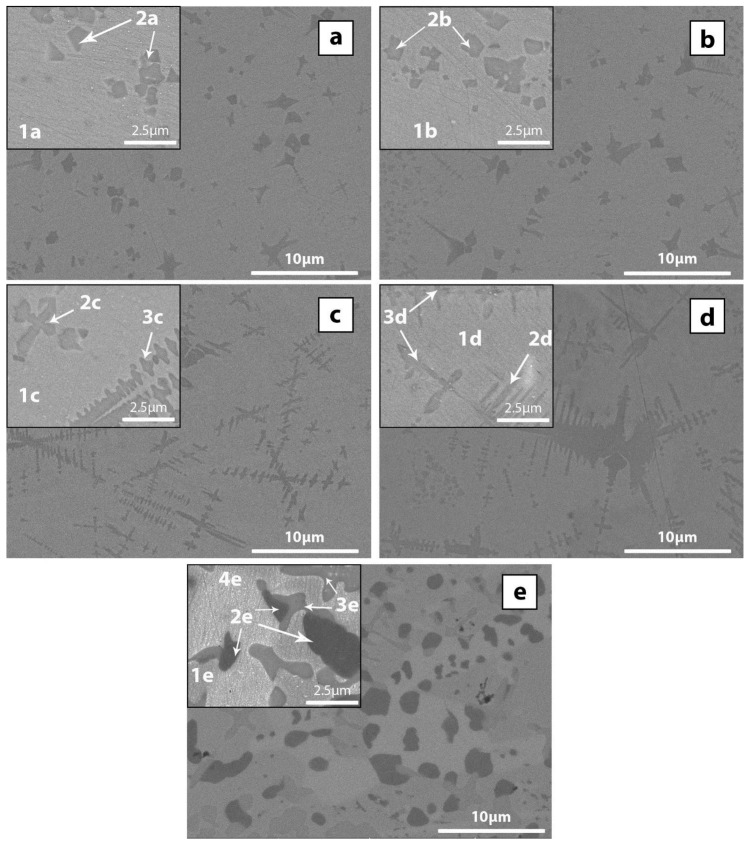
Backscattered SEM images taken from the middle radius for Cu_52_Zr_41_Al_7_ at. % alloy (**a**) CH 2 mm diameter; (**b**) NCH 2 mm diameter; (**c**) CH chilled 3 mm diameter; (**d**) NCH 3 mm diameter; (**e**) 3 mm crystallised sample.

Figure 2 shows the backscattered SEM images taken from the middle radius of the rod´s cross section obtained at different cooling rates and also for the crystallised rod. The microstructure consists of crystalline phases embedded in a featureless amorphous matrix, which is consistent with the XRD results. For the CH 2 mm sample (Figure 2a), crystalline phases of round and irregular (dendrite-like) shape of up to 2 μm size can be detected. The contrast of the crystalline phases is practically the same and therefore a single crystalline phase seems to be present with a constant composition. The darker appearance of the crystalline phase compared to the matrix suggests that this phase has a lower atomic weight than the matrix. This phase may correspond to CuZr since the Zr (the heaviest element) to Cu ratio is larger than that of the nominal composition. For the NCH 2 mm diameter sample (Figure 2b), slight differences can be observed. Crystalline phases of round and irregular (dendrite-like) shape are also present in this sample; the size of the irregular phase is slightly larger and can reach up to about 4 μm. The darker circular dendritic phases may be associated with CuZr.

The morphology of the crystalline phase for the CH 3 mm sample (Figure 2c) changes dramatically as well-developed dendrites are observed across the sample. The arms of these dendrites can reach up to 10 μm length and some of them are partly connected. A higher density of crystalline phases can be observed for the NCH 3 mm sample (Figure 2d) for which connectivity between the well-developed crystalline dendrites (up 18 μm in length between arms) starts to appear. This sample exhibits both the light and dark intermetallic phases as discussed for the previous sample. Similar phenomena have been observed during the crystallisation process for different alloy compositions where one crystalline phase grows from the previously formed phase [41]. For the annealed alloy (Figure 2e), multiple phases seem to be present. Dark geometric particles up to 5 μm can be found surrounded by grey dendrites of smaller size (up to 4 μm) and a lighter-coloured phase.

To analyse in more detail the phases present for each composition, the microstructures at higher magnification have been observed (backscattered SEM images of Figure 2). The composition of each phase was analysed by EDX (always given in at. %) and the results are listed in Table 1. For the CH 2 mm (inset of Figure 2a) and NCH 2 mm alloy (inset of Figure 2b) the composition of the crystalline phase is Cu_43.3_Zr_44.9_Al_11.9_ (phase 2a) and Cu_45.6_Zr_43.6_Al_10.7_ (phase 2b), respectively with Cu/Zr ~ 1 and therefore both phases could correspond to CuZr, which has been frequently detected for similar compositions [42,43]. The composition of the amorphous matrix is very similar for the CH and NCH samples, Cu_51.2_Zr_43.1_Al_6.8_ (phase 1a) and Cu_51.4_Zr_42.5_Al_6.3_ (phase 1b), respectively, thus they are close to the nominal composition (i.e., Cu_52_Zr_41_Al_7_) of the alloy.

For the CH 3 mm (inset of Figure 2c) and NCH 3 mm samples (inset of Figure 2d) two different crystalline phases are detected according to the EDX results, an increase in size of the dendrites can be observed, reaching up to 10 µm in size. Two different crystalline phases were found in this sample. The composition of the first one for the CH is Cu_45.6_Zr_43.0_Al_11.4_ (phase 2c) and Cu_45.9_Zr_43.0_Al_11.1_ (phase 2d) for the NCH. Having similar concentration in Cu and Zr suggests that this phase corresponds to CuZr. The secondary crystalline phases have larger dimensions and their compositions are Cu_37.7_Zr_48.3_Al_13.9_ (phase 3c) and Cu_37.3_Zr_49.4_Al_13.5_ (phase 3d). The Cu/Zr ratio for both phases is about 0.78 suggesting that they may correspond to CuZr_2_ [42,44,45]. The composition of the matrix is Cu_52.0_Zr_42.1_Al_5.9_ (phase 1c) and Cu_52.5_Zr_42.1_Al_7.2_ (phase 1d), for the CH and NCH alloys, both similar to the nominal composition. Surrounding these crystalline phases, a slight discolouration seems to be present. Nevertheless, due to its small size it has proven difficult to differentiate this phase from the matrix. It has to be noticed that in our previous studies the same decolouration was found to be correlated with the presence of Cu_10_Zr_7_ [25]. For the crystallised alloy (inset of Figure 2e), the magnified image enables a better appreciation of the differences in contrast between the crystalline phases and the general image; it is possible to observe an additional phase. For the round-shaped darkest phase (phase 2e), the EDX results indicate that its composition is Cu_54.8_Zr_28.5_Al_16.7_ and thus it could be attributed to the ternary Cu_2_ZrAl phase. The dendrites near the geometric particles can be associated with CuZr_2_ since their composition is Cu_36.7_Zr_51.02_Al_13.8_ (phase 3e). With regard to the two crystalline phases surrounding the commented phases, compositional differences were found between the lighter and the darker phases. Similar compositions were found for the two crystalline phases. On the one hand, the darker crystalline phase revealed a composition of Cu_54.9_Zr_43.3_Al_1.9_ (phase 1e). On the other hand, the lighter phase was characterised by higher Zr content with a composition of Cu_46.8_Zr_51.0_Al_2.2_ (phase 4e). As a result, the darker phase may be correlated with Cu_10_Zr_7_, while the clearer phase reveals a composition near CuZr.

### 3.2. Scratch Tests

**Figure 3:**
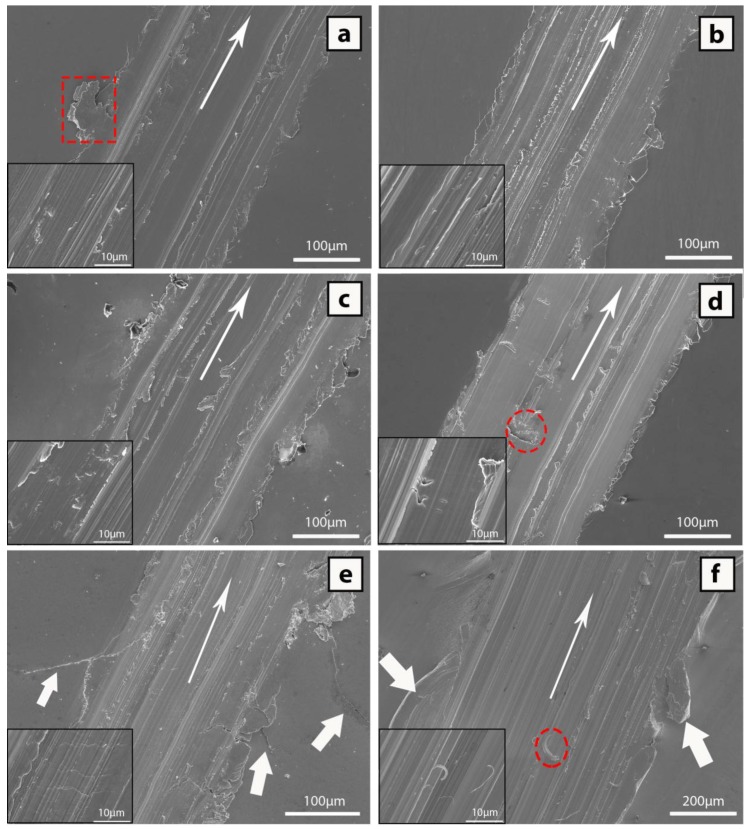
Images showing the scratches at the middle radius of the samples (**a**) CH 2 mm diameter; (**b**) NCH 2 mm diameter; (**c**) CH 3 mm diameter; (**d**) NCH 3 mm diameter; (**e**) 3 mm annealed at 850 °C for 48 h; (**f**) 3 mm pure copper.

**Figure A1:**
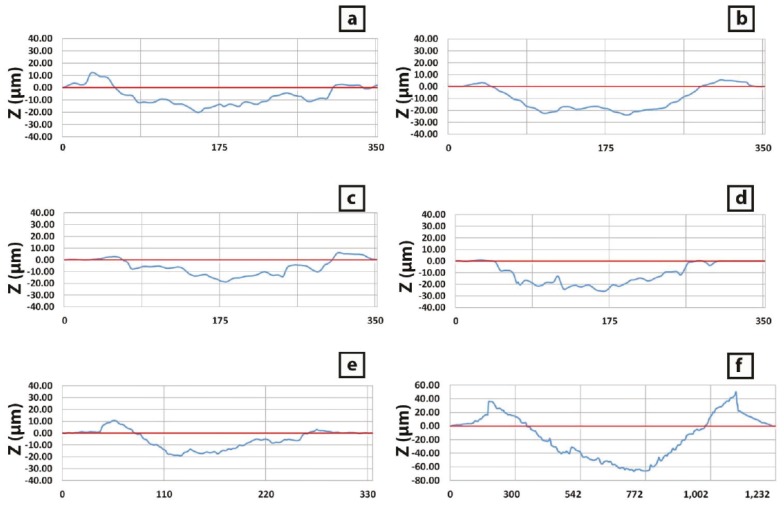
Graphs showing the scratches at the middle radius of the samples (**a**) CH 2 mm diameter; (**b**) NCH 2 mm diameter; (**c**) CH 3 mm diameter; (**d**) NCH 3 mm diameter; (**e**) 3 mm annealed at 850 °C for 48 h; (**f**) 3 mm pure copper.

In order to assess the wear behaviour of the samples, scratch tests were performed from 10 different measurements at approximately half the radius distance from the centre of the rods. Differences in wear behaviour were defined from the morphology of the scratches (Figure 3) and from the cross section profile (Figure A1). For each composition, the pile-up size, groove depth at the centre and maximum groove depth, groove area over the pile-up area, the track width and the scratch hardness number are listed on Table 2. Considering the low sliding speed (10 mm/min) and load (30 N) applied during the test, any temperature rise caused by the frictional force between the diamond tip and the substrate is not expected to be enough to result in crystallisation [46].

#### 3.2.1. CH 2 and NCH 2 Samples

Figure 3a shows the scratch for the CH 2 mm sample. The morphology of the scratch consists of relatively high pile-up region with dispersed large “volumes” of material removed from the groove and adhered to the sides of the track (dashed square). The pile-up height is 5.05 ± 1.92 μm while the groove depth at the centre and the maximum depth are 15.70 ± 1.22 μm and 20.79 ± 1.41 μm, respectively (Table 2). The high pile-up suggests that the stresses created by the diamond tip results in considerable plastic deformation. Long continuous lines of smeared material distributed along the centre of the track are observed; these are typical features of adhesive wear in ductile materials. Long narrow grooves within the track are evidence of abrasive wear from hard phases adhered to the diamond tip. These features were not observed in metallic glass composites of similar composition corresponding to the Cu-Zr-Al system when tested at the same conditions [25]. These continuous lines of smeared material suggest that the Cu_52_Zr_41_Al_7_ alloy is more ductile than those previously reported [25] and shows that the mechanical performance (ductility/brittleness) is very composition dependent. The scratch width of this sample is 243.16 ± 2.36 μm and therefore, from Equation (1), the scratch hardness value is 1.29 ± 0.02 GPa.

For the NCH 2 mm sample (Figure 3b) an increase in smearing can be deduced from the groove track compared to the CH 2 mm sample. This smearing is dominated by long (up to 300 µm) and thin (10 µm) lines of material. As the matrix of the material is more prone to wear than the hard particles embedded in it, the removed material acts as a secondary cause of wearing by abrasion besides to the direct interaction between substrate and tip. The density of lines is higher and the height of the pile up (Figure A1) is smaller for the NCH 2 mm sample, which is consistent with the slightly larger volume fraction of brittle intermetallic phases for NCH 2 mm (Figure 1 and Figure 2). The height of the pile up decreases substantially to about 3.71 ± 1.58 µm, while the depth at the centre (17.46 ± 0.85 μm) and at the maximum depth (21.82 ± 1.45 μm) is larger than that of the CH 2 mm sample. The scratch hardness number increases from 1.29 for CH 2 mm to 1.31 for NCH 2 mm due to the presence of a relatively large volume fraction of brittle intermetallic phases.

#### 3.2.2. CH 3 and NCH 3 Samples

For the CH 3 mm sample (Figure 3c), the smearing is more prominent and the height of the pile-up is slightly smaller than for the 2 mm diameter samples (Figure 3a,b). The height of the pile-up decreases to 2.78 ± 1.09 μm but the groove depth at the centre (17.48 ± 1.79 μm) and the maximum depth (19.82 ± 2.22 μm) barely change. The continuous lines of smeared material and grooves are probably caused by the constrained (squeezing) soft amorphous phase located between the large crystalline particles. The amorphous matrix is thus subjected to large plastic deformation, which can explain the formation of shear bands (dashed ellipses) that were not observed for the CH and NCH 2 mm samples. From the track width of the sample, 240.30 ± 2.98 μm, a scratch hardness of 1.32 ± 0.03 GPa is obtained, similar to the value for NCH 2 mm.

Finally, for the NCH 3 mm sample (Figure 3d) smearing features along with randomly distributed large (dashed circle) and small pits (see inset) along the track were observed. These pits are larger (~ 50 μm) and less abundant than those previously reported for Cu_56_Zr_38.7_Al_5.3_ alloy [25], which can be attributed to differences in microstructure. The pits are probably caused by the reaction of the Zr-containing intermetallic particles with the carbon from the diamond tip, which results in sticking and pulling out of the particles. This might be confirmed by the fact that the pits are observed at the centre of the track, where direct contact with the tip takes place, rather than at the outer side, where the contact is mostly with the dragged particles. The sticking efficiency is expected to be the highest for the CuZr_2_ dendrites. These dendrites, however, are smaller than 50 μm (the pit size), which suggests that not only CuZr_2_ is being pulled out but probably also CuZr and the matrix. The cross section profile (Figure A1) shows a slight decrease in pile-up height, i.e., 2.63 ± 1.51 μm, but a substantial increase in groove depth at the centre, i.e., 21.92 ± 1.83 μm, and maximum groove depth, i.e., 26.51 ± 2.42 μm. The small pile-up height suggests embrittlement, which agrees with the increase in volume fraction of brittle intermetallic particles detected from the XRD scan (Figure 2). The increase in hardness caused by the increase of Cu-rich crystal phases are in accordance with the track width and scratch hardness values shown in Table 2. The alloy with the largest volume fraction of crystalline phases exhibits a much lower width track (229.44 ± 5.99 µm) and higher scratch hardness number (1.45 ± 0.08 GPa), which is also in accordance with the microstructure.

In summary, the change in wear behaviour for the metallic glass composites is mostly attributed to the increase in volume fraction of crystalline phases (estimated from the differences in tonality of the backscattered SEM images). The volume fraction was about 20%, 25%, 30% and 50% for the CH 2, NCH 2, CH 3 and NCH 3 samples, respectively. For the first three samples, the percolation threshold (the volume fraction at which connectivity of the crystalline phases first takes place) is not reached [47]. However, once the percolation threshold is reached (only for NCH 3 alloy), a network of crystalline phases is formed, causing an abrupt change in the mechanical properties [48], which is consistent with the rapid increase of the scratch hardness number from 1.32 to 1.45.

#### 3.2.3. Annealed and Pure Copper Samples

In order to assess the wear performance of the material in a fully crystallised state, the sample was annealed at 850 °C for 48 h (Figure 3e). The height of the pile up is 2.90 ± 1.31 μm and there is a large variation in the pile-up height along the scratch, with most sites having a very low height but also local sites of high pile-up present. However, the values of groove depth (17.47 ± 1.13 μm) and maximum groove depth (20.74 ± 1.22 μm) are similar to the NCH 2 mm and CH 3 mm samples. The scratch width is the lowest of all the analysed samples (196.08 ± 1.57 μm), for which a scratch hardness number of 1.99 ± 0.03 GPa is obtained. This hardness number is larger than for the metallic glass composites, which is consistent with the microstructural differences. The most noticeable feature of this sample is the cracks located at both sides of the scratch (white arrows). These cracks propagate from the sides of the scratch for a distance of about 100 μm. The magnified image from the centre of the scratch (inset) shows that the surface of the groove is smooth and the presence of small cracks of up to 15 μm propagating transversally to the scratch direction. Due to the lack of a relatively ductile amorphous matrix that would enable the particles to be smeared, the stresses generated upon scratching can only be released through the formation of cracks. The low pile-up, groove depth and scratch width along with the presence of cracks are features consistent with the higher hardness and wear resistance than the metallic glass composites.

For comparison purposes scratch tests were carried out at the middle radius on pure copper (Figure 3f). The general image of Figure 3f shows a track width of 426.54 ± 3.61 µm, much larger than that of the Cu_52_Zr_41_Al_7_ sample, which is consistent with the low scratch hardness values obtained, i.e., 0.42 ± 0.01 GPa, using equation 1. The height of the pile-up (white arrows) is 40.91 ± 12.08 µm, much higher than any of the studied samples for Cu_52_Zr_41_Al_7_, which again confirms the low wear resistance of copper. At the same time, the groove centre shows a uniform surface exhibiting some smearing with groove depth and maximum groove of 61.93 ± 3.97 µm and 66.34 ± 2.92 µm, respectively. One interesting feature that must be mentioned is the hooked structures present in the centre of the track, which are typical features of a plastically deformed material (inset).

Comparing, the height of the pile-up, the ratio of the groove area to pile-up area and the scratch hardness values (Table 2) for all the analysed samples, one can conclude that the scratch hardness provides the most reliable results for assessing the wear performance. An important factor is that the standard deviation error for scratch width measurements is at least one order of magnitude smaller than the error when measuring the pile-up and groove.

### 3.3. Wettability and Antimicrobial Tests

**Figure A2:**
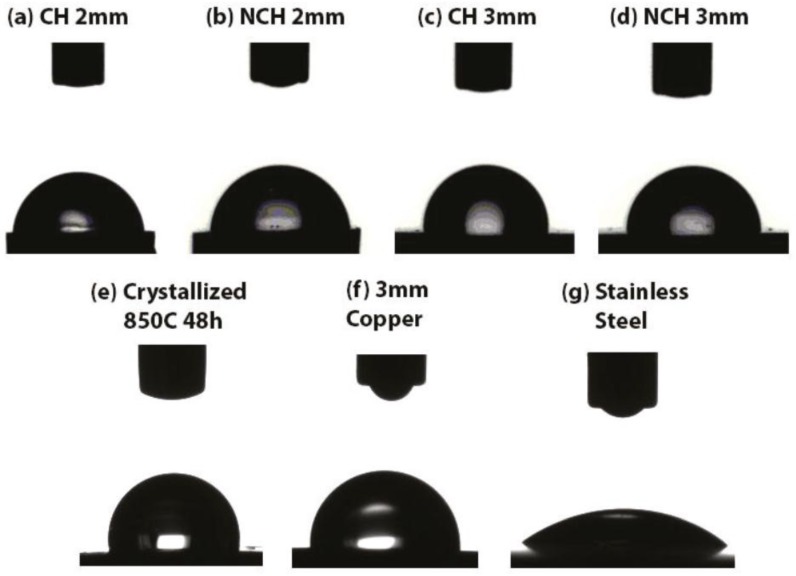
Average water contact angle Cu52Zr41Al7 at. % alloy (**a**) CH 2 mm diameter; (**b**) NCH 2 mm diameter; (**c**) CH 3 mm diameter; (**d**) NCH 3 mm diameter; (**e**) Crystallized at 850 °C for 48 h; (**f**) 3 mm pure copper; (**g**) stainless steel.

In order to evaluate the use of these copper-based alloys for antimicrobial purposes, their wettability was studied. This parameter can be used to predict the attachment of bacteria to contact surfaces [49]. Wettability can be evaluated by measuring the contact angle of sessile droplets (Figure A2). Results revealed an average water contact angle (deionised water) of 88.6 ± 1.1° on the CH 2 mm diameter, 91.9 ± 1.5° on the NCH 2 mm diameter, 96.7 ± 1.4° on the CH 3 mm diameter and 96.2 ± 2.7° on the NCH 3 mm diameter. As a reference, the contact angle of a Cu_52_Zr_41_Al_7_ 3 mm rod annealed at 850 °C for two days, a 3 mm pure copper rod and stainless steel plate were measured, obtaining 94.6 ± 5.1°, 93.9 ± 4.5° and 41.5 ± 4.6° respectively. Greater differences can be appreciated between the 2 mm samples rather than the 3 mm samples, which is consistent with the structural changes revealed by the XRD scans. The change in microstructure results in samples with increasing hydrophobic properties. It is interesting to notice that the 3 mm crystallised sample reached a value similar to the one obtained for pure copper which might be explained by the increase in Cu-rich phases. This increase of contact angle as the microstructure evolves suggests that bacteria would be less likely to attach to more crystalline surfaces. Consequently, tuning the volume fraction of amorphous and crystalline phase in metallic glass composites has the potential to be used to control the antimicrobial behaviour.

**Figure 4:**
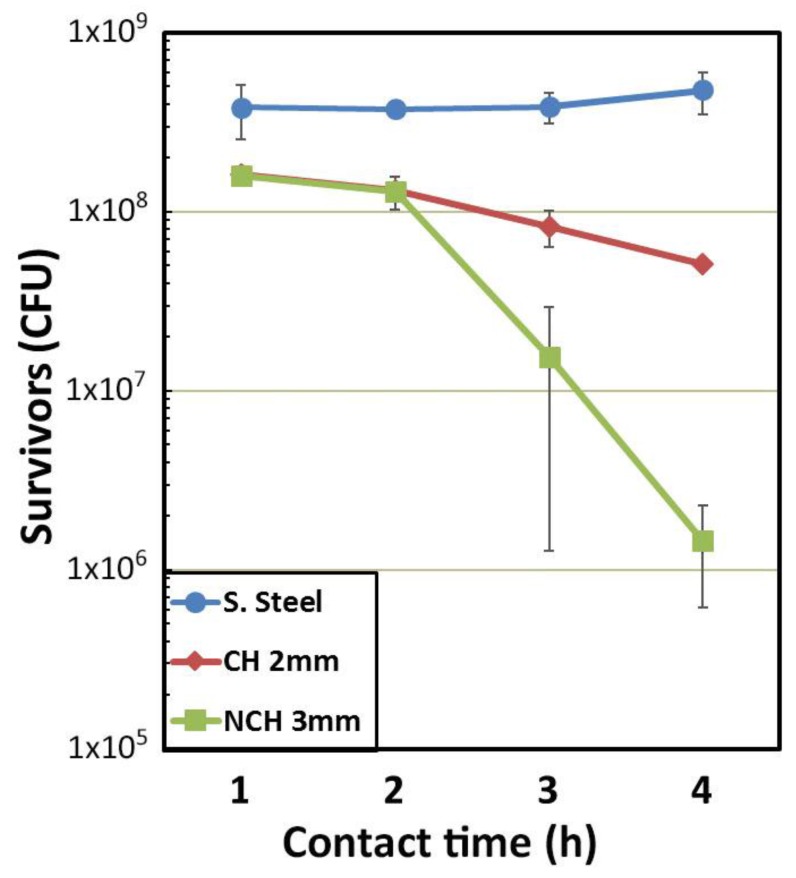
Recovery of *E. coli* exposed to the indicated substrates. Each surface was inoculated with 1 µL of bacterial culture grown until OD_600_ = 0.3.

**Figure 5:**
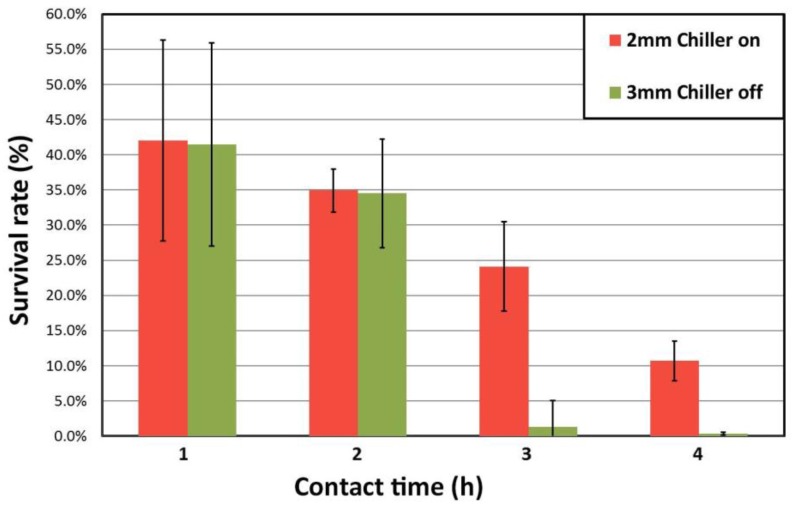
Survival rate values for samples with highest difference in cooling rate (2 mm chiller and 3 mm no chiller) after 1, 2, 3 and 4 h using the Stainless Steel CFU as 100% survival ratio.

The antibacterial activity of the alloys was assessed by following the reduction in recovered *E. coli* colony-forming units (CFU) from the surface over time. Figure 4 shows the number of *E. coli* CFU for the stainless steel and the two samples with the greatest disparity in cooling rate (CH 2 mm and NCH 3 mm). The stainless steel coupons maintain an almost constant amount of bacteria (≈ 3.8 × 10^8^ CFU/mL) up to 4 h; for this reason, they have been used as a reference for calculation of the bacteria survival percentage. In contrast, the Cu-containing samples exhibit antimicrobial behaviour after 1 h of inoculation. No large differences are evident during the first two hours of interaction between the bacteria and these Cu-containing samples. However, for longer times, i.e., 3 and 4 h, differences in CFU values were observed. The NCH 3 mm sample seems to be more active than the CH 2 mm sample, with recovered populations almost one order of magnitude lower than the CH 2 mm sample. Figure 5 shows the survival rate for the same samples after 1, 2, 3 and 4 h of contact time. The calculation has been done using the equation reported by Chu et al. [23] but expressed as the survival rate:

(2) $$\mathrm{Survival}\;\mathrm{rate}=1-\frac{{\mathrm{CFU}}_{S.\mathrm{Steel}}-{\mathrm{CFU}}_{\mathrm{Sample}}}{{\mathrm{CFU}}_{S.\mathrm{Steel}}}\;,$$

where CFU_S.Steel_ and CFU_Sample_ are the recovered *E. Coli* colony-forming units from stainless steel and the sample, respectively. The percentage of bacteria eliminated after 1 h corresponds to 58% of the bacteria recovered from the stainless steel coupons and the values for CH 2 mm and NCH 3 mm samples are similar. A similar trend was observed after two hours of contact killing: both samples were able to eliminate almost 65% of the recoverable bacteria, but for the NCH 3 mm sample the antimicrobial activity was slightly higher. A marked change seems to occur after 3 h, when a dramatic increase in the number of *E. coli* eliminated is observed, reaching 75.9% for the CH 2 mm alloy and 98.9% for the NCH 3 mm alloy. Finally, after 4 h only 0.3% of bacteria remain on the NCH 3 mm surface, while the CH 2 mm sample shows a survival ratio of 10.7%. These results suggest that CH 3 mm is the most interesting material for healthcare purposes. For comparison purposes, the same experiment was conducted on the fully crystallised Cu_52_Zr_41_Al_7_ alloy. After one hour of contact killing, only 17.3% of bacteria were able to survive on this surface and no bacteria were detected after two hours of inoculation. The last samples showed cracks and a partial loss of material, probably attributed to intergranular corrosion. This may explain the fast elimination of bacteria since Cu ions, which are regarded as responsible for the antimicrobial behaviour, can be easily released during corrosion.

Evolution of the *E. Coli* survival rate with the contact time for up to 4 h for CH 2 mm sample follows approximately a linear trend, while for the NCH 3 mm sample the survival rate drops dramatically after 3 h, suggesting a change in the kinetics. This difference in antibacterial activity might be attributed to the piling of cell dead biomass on the surface of the sample. It was reported that several mechanisms would be responsible for the contact killing properties of copper [14,50]. However, the accumulation of Cu ions on the surface of cell membranes also has an important role [17,51]. During the first hours of contact killing, the dead and damaged biomass would be piled on top of the surface, offering protection to individual *E. Coli* cells. As a result, the antibacterial activity of the samples is halted. The increase in Cu ion concentration over the contact time would be able to overcome the protective film, thus resulting in an increase in antibacterial activity. The difference between the CH 2 mm and NCH 3 mm sample could be explained by the mobility of ions promoted by larger grain boundaries. As the samples become more crystalline, the number of grain boundaries increases. This leads to a larger density of copper ions that can be released, since grain boundaries are easy paths for ion diffusion [52]. The more crystalline NCH 3 mm sample exhibits more potential diffusion paths than the less crystalline NC 2 mm sample and therefore the protective layer of the former sample would be more easily overcome. The reduction of *E. Coli* colony-forming units during the formation of the first protective layer would prevent the development of a new film, thus leading to the observed bactericidal effect.

For the crystallised sample after annealing, Cu_10_Zr_7_ and Cu_2_ZrAl were found. The increase of ion diffusion caused by the grain boundaries linked to the presence of Cu-rich phases might explain the high bactericidal effect of this sample. Interestingly, it might be apparent that the annealed sample would be of interest for antimicrobial applications. Nevertheless, after the antimicrobial experiments this sample showed signs of corrosion and brittleness, which might influence the antimicrobial effect due to the appearance of copper oxide [53,54]. Corrosion and embrittlement were not observed in the metallic glass composites. As a result, metallic glass composites can be regarded as interesting candidates for antimicrobial applications.

It has been shown that standard methodologies exist in regard to the evaluation of the antimicrobial surfaces such as Japanese standard “Antibacterial products-Test for antibacterial activity and efficacy” [31], the European “Plastics-Measurement of antibacterial activity on plastic surfaces” [32] and the American “Protocol for the evaluation of bactericidal activity of hard, non-porous copper-containing surface products” [33]. These documents relay methods, guidelines and equations for antibacterial activity assessment. It should be noted that in this research the inoculum populations are much higher than those described in the standards (6 × 10^5^ CFU/mL in the Japanese standards and 4–5 logs/carrier in the EPA standard). However, higher concentrations of bacteria are commonly used in the measurement bactericidal properties of metallic glasses, 10^5^–10^6^ CFU/mL [23,55] or OD_600_ = 0.3 [24]. To analyse the impact of standardisation on our dataset, two equations available in the aforementioned standards were used to estimate the antimicrobial activity (Table 3 and Table 4) of our samples. Equation 3 [31] calculates the antimicrobial activity as:

(3) $$R=\left(U_{i}-\;U_{0}\right)-\left(A_{i}-U_{0}\right)=U_{i}-\;A_{i}\;,$$

where *R*, *U_i_*, *U_0_*, and *A_i_* refer to antimicrobial activity, average of logarithm numbers of viable bacteria immediately after inoculation on untreated test pieces, average of logarithm numbers of viable bacteria immediately after inoculation on untreated test pieces after 24 h and average of logarithm numbers of viable bacteria immediately after inoculation on antibacterial test pieces after 24 h, respectively.

On the other hand, Equation 4 [33] makes use of the geometric mean of the colony-forming units as:

(4) $$\%\;reduction=\;\frac{a-b}{a}*100,$$

where *a* and *b* refer to the geometric mean of the number of organisms surviving on the inoculated control carriers and the geometric mean of the number of organisms surviving on the test carriers, respectively. There is a slight difference between the results shown in Figure 5 and the data calculated in Table 4. The results shown in Figure 5 have been calculated as the arithmetic mean of the test results. On the other hand, Table 4 was obtained using the geometric mean as defined in the USA EPA Protocol for the Evaluation of Bactericidal Activity of Hard, Non-Porous Copper-Containing Surface Products.

Due to the origin of the equations, it is not possible to compare the methods. Nevertheless, the same trend can be seen in both cases. As the contact time increases, the antimicrobial activity and the percentage of reduced bacteria increase, reaching a maximum of 2.51 antimicrobial activity or 99.91% of bacteria reduction percentage for the NCH 3 mm sample. This value is close to the 3-log reduction threshold provided by the American protocol to consider an antimicrobial surface as effective [33]. Due to the amount of bacteria in the inoculum (≈ 3.8 × 10^8^ CFU/mL) a reduction of 99.9% of bacteria (≈ 3.8 × 10^5^ CFU/mL) might not be enough to ensure the safety of a patient. The results reveal that the metallic glass composites analysed might not be considered as antibacterial surfaces using the provided standards. However, this material shows interesting properties, and more in-depth research must be conducted to optimise the antibacterial effect.

## 4. Conclusions

From the study of the effect of cooling rate on the performance of the materials studied, the following conclusions can be drawn:

1. For Cu_52_Z_41_Al_7_ alloy composition, a decrease of the cooling rate from the melt results in an increase in the wear resistance, linked to the embrittlement of the samples, as deduced from the scratch tests. This is revealed in the lower pile-up, higher groove, prone adhesion wear and increase in scratch hardness from 1.29 GPa for sample CH 2 mm to 1.45 GPa for sample NCH 3 mm.
2. The sessile drop test shows an increase in contact angle as the microstructure gets more crystalline. As a result, adhesion of bacteria is less likely to occur in higher crystalline composites.
3. The cooling system that keeps the mould cold (chiller on: 10 °C and off: 20 °C) upon suction casting has little effect on the microstructure and therefore on the alloy performance compared to the effect of the mould cavity diameter. The effect of using the chiller is practically negligible for the 2 mm diameter samples and becomes slightly larger for the 3 mm diameter samples.
4. Rapid solidification has been proven to be an efficient technique to tune the properties of the Cu-Zr-Al system upon cooling. The rapid quenched Cu_52_Z_41_Al_7_ alloy can be used as a precursor to tune the microstructure upon annealing (i.e., 850 °C for 48 h) and reach an antimicrobial performance beyond the threshold provided by the American protocol (i.e., the alloy is made antimicrobial). Annealing the alloy also enables us o improve the wear resistance by increasing the scratch hardness from 1.45 to 1.99 GPa.
5. The crystalline sample obtained by annealing at 850 °C for 48 h exhibits the best performance in terms of antimicrobial behaviour and wear resistance (i.e. scratch hardness of 1.99 ± 0.03 GPa). However, the sample corrodes very easily and breaks into small pieces after only one hour of inoculation in sterile LB broth due to the large number of grain boundaries and the nature of the crystalline phases.
6. The 3 mm diameter Cu sample exhibits a scratch hardness value of 0.4 GPa, more than three times lower than that of the 3 mm NCH metallic glass composite. Therefore, depending on the application (antimicrobial medical devices, touch surfaces, etc.), a compromise between durability and antimicrobial performance is needed and a composite might be preferred over pure copper.

## Figures and Tables

**Table 1:** Composition in at. % of the areas labelled on Figure 2 and phases to which they can be attributed.

Element	1a matrix	1b matrix	1c matrix	1d matrix	2a Dendrites I	2b Dendrites I	2c Dendrites I	2d Dendrites I	3c Dendrites II	3d Dendrites II	1e matrix	2e Geometric Particles	3e Dendrites	4e Clear Matrix
Cu	51.2 ± 0.2	51.1 ± 0.4	52.1 ± 0.4	52.5 ± 0.6	43.3 ± 3.0	45.6 ± 0.8	45.6 ± 1.0	45.9 ± 1.5	37.7 ± 0.1	37.3 ± 1.3	54.9 ± 1.0	54.8 ± 1.3	36.7 ± 1.5	46.8 ± 0.7
Zr	43.2 ± 3.3	42.6 ± 0.2	42.1 ± 1.9	40.4 ± 0.5	44.9 ± 1.8	43.6 ± 1.8	43.0 ± 0.7	43.0 ± 1.2	48.3 ± 0.2	49.4 ± 1.0	43.3 ± 0.9	28.5 ± 1.7	49.6 ± 0.9	51.1 ± 0.5
Al	6.8 ± 0.2	6.3 ± 0.4	5.85 ± 1.7	7.15 ± 0.7	11.9 ± 1.4	10.7 ± 1.2	11.4 ± 0.7	11.1 ± 0.7	13.9 ± 0.2	13.46 ± 0.5	1.9 ± 0.3	16.7 ± 1.2	13.8 ± 1.6	2.2 ± 0.6
Phase	Nominal comp.	Nominal comp.	Nominal comp.	Nominal comp.	CuZr	CuZr	CuZr	CuZr	CuZr_2_	CuZr_2_	Cu_10_Zr_7_	Cu_2_ZrAl	CuZr_2_	CuZr

**Table 2:** Summary of the pile-up height, groove deep at the centre and maximum, groove area over the pile-up area, track width and scratch hardness number for Cu_52_Zr_41_Al_7_ alloy (**a**) CH 2 mm diameter; (**b**) NCH 2 mm diameter; (**c**) CH 3 mm diameter; (**d**) NCH 3 mm diameter; (**e**) 3 mm crystallised sample.

Sample	Pile-Up (µm)	Centre (µm)	Maximum (µm)	Groove Area/ Pile-Up Area	Scratch Width (µm)	Scratch Hardness Number (GPa)
CH 2 mm	5.05 ± 1.92	15.70 ± 1.22	20.79 ± 1.41	10.05 ± 3.87	243.16 ± 2.36	1.29 ± 0.02
NCH 2 mm	3.71 ± 1.58	17.46 ± 0.85	21.82 ± 1.45	14.51 ± 5.87	241.25 ± 2.23	1.31 ± 0.02
CH 3 mm	2.78 ± 1.09	17.48 ± 1.79	19.82 ± 2.22	17.20 ± 3.72	240.30 ± 2.98	1.32 ± 0.03
NCH 3 mm	2.63 ± 1.51	21.92 ± 1.83	26.51 ± 2.42	31.83 ± 5.90	229.44 ± 5.99	1.45 ± 0.08
850 °C 48 h	2.90 ± 1.31	17.47 ± 1.13	20.74 ± 1.22	13.97 ± 6.75	196.08 ± 1.57	1.99 ± 0.03
Copper	40.91 ± 12.08	61.93 ± 3.97	66.34 ± 2.92	2.97 ± 0.88	426.54 ± 3.61	0.42 ± 0.01

**Table 3:** Antimicrobial activity (R) calculated using JIS Z 2801:2010 Antibacterial products—test for antibacterial activity and efficacy.

Sample	Parameter	1 h	2 h	3 h	4 h
CH 2 mm	*Ui*	8.58	8.57	8.59	8.68
	*Ai*	8.21	8.12	7.92	7.71
	***R***	**0.38**	**0.46**	**0.67**	**0.97**
NCH 3 mm	*Ui*	8.58	8.57	8.59	8.68
	*Ai*	8.20	8.11	7.19	6.16
	***R***	**0.38**	**0.46**	**1.40**	**2.51**

**Table 4:** Reduction of bacteria percentage calculated using the U.S. EPA Protocol for the Evaluation of Bactericidal Activity of Hard, Non-Porous Copper-Containing Surface Products.

Sample	Parameter	1 h	2 h	3 h	4 h
CH 2 mm	a (control)	2.62 × 10^8^	3.14 × 10^8^	3.25 × 10^8^	4.60 × 10^8^
	*b*	1.61 × 10^8^	1.74 × 10^8^	2.24 × 10^7^	2.68 × 10^7^
	**% reduction**	**38.73%**	**44.57%**	**93.09%**	**94.18%**
NCH 3 mm	a (control)	2.62 × 10^8^	3.14 × 10^8^	3.25 × 10^8^	4.60 × 10^8^
	*b*	1.59 × 10^8^	1.78 × 10^8^	9.44 × 10^5^	4.23 × 10^5^
	**% reduction**	**39.52%**	**43.22%**	**99.71%**	**99.91%**

## Appendix A

Supplementary Figure A1 shows the cross section profile of the scratch test for the CH 2 mm, NCH 2 mm, CH 3 mm, NCH 3 mm, the sample annealed at 850 °C for 48 h and the 3 mm pure copper samples. The horizontal line at z = 0 corresponds to the substrate level and it is used as reference to measure the pile-up height (maximum) and groove depth, i.e., the depth of the track at the centre and maximum depth of the track. In this figure, the change in pile-up height and groove depth can be appreciated as the cooling rate decreases. It is of interest to notice the large scratch width, pile-up height and groove depth for the pure copper sample.

Supplementary Figure A2 displays the contact angle for the CH 2 mm, NCH 2 mm, CH 3 mm, NCH 3 mm, the sample annealed at 850 °C for 48 h, the 3 mm pure copper and the stainless steel samples. A subtle increase in contact angle can be detected as the cooling rate decreases, thus making it more difficult for bacteria to adhere onto the sample’s surface. Interestingly, the results showed by the annealed and pure copper samples stand between the 2 mm and 3 mm BMG composites. An increase in crystallinity upon cooling leads to higher contact angle values and therefore would decrease the adhesion of bacteria.
